# User's perspectives of barriers and facilitators to implementing quality colonoscopy services in Canada: a study protocol

**DOI:** 10.1186/1748-5908-5-85

**Published:** 2010-11-02

**Authors:** Gilles Jobin, Marie Pierre Gagnon, Bernard Candas, Catherine Dubé, Anis Ben Abdeljelil, Sonya Grenier

**Affiliations:** 1 Department of Medicine, Université de Montréal, Montréal, Canada; 2 Maisonneuve-Rosemont Hospital, Montréal, Canada; 3 Department of Nursing, Université Laval, Québec, Canada; 4 Research Center of the Centre Hospitalier Universitaire de Québec, Québec, Canada; 5 Department of Medicine, Université Laval, Québec, Canada; 6 Canadian Partnership Against Cancer, Québec, Canada; 7 University of Calgary, Calgary, Alberta, Canada

## Abstract

**Background:**

Colorectal cancer (CRC) represents a serious and growing health problem in Canada. Colonoscopy is used for screening and diagnosis of symptomatic or high CRC risk individuals. Although a number of countries are now implementing quality colonoscopy services, knowledge synthesis of barriers and facilitators perceived by healthcare professionals and patients during implementation has not been carried out. In addition, the perspectives of various stakeholders towards the implementation of quality colonoscopy services and the need of an efficient organisation of such services have been reported in the literature but have not been synthesised yet. The present study aims to produce a comprehensive synthesis of actual knowledge on the barriers and facilitators perceived by all stakeholders to the implementation of quality colonoscopy services in Canada.

**Methods:**

First, we will conduct a comprehensive review of the scientific literature and other published documentation on the barriers and facilitators to implementing quality colonoscopy services. Standardised literature searches and data extraction methods will be used. The quality of the studies and their relevance to informing decisions on colonoscopy services implementation will be assessed. For each group of users identified, barriers and facilitators will be categorised and compiled using narrative synthesis and meta-analytical techniques. The principle factors identified for each group of users will then be validated for its applicability to various Canadian contexts using the Delphi study method. Following this study, a set of strategies will be identified to inform decision makers involved in the implementation of quality colonoscopy services across Canadian jurisdictions.

**Discussion:**

This study will be the first to systematically summarise the barriers and facilitators to implementation of quality colonoscopy services perceived by different groups and to consider the local contexts in order to ensure the applicability of this knowledge to the particular realities of various Canadian jurisdictions. Linkages with strategic partners and decision makers in the realisation of this project will favour the utilisation of its results to support strategies for implementing quality colonoscopy services and CRC screening programs in the Canadian health system.

## Background

Colorectal cancer (CRC) is currently seen as a serious and growing public health problem in Canada [1]. CRC is the third most common type of cancer in Canadian men and women. In 2010, 9,100 persons are expected to die from CRC, making it the second most lethal cancer for Canadians [1]. Several randomised controlled trials have supported the fact that screening with a faecal occult blood test significantly reduces CRC-related mortality [2,3], and many countries have implemented screening programs using this test [4]. Moreover, a large, randomised, controlled trial conducted in the United Kingdom showed that one flexible sigmoidoscopy screen performed between the ages of 55 and 64 years old can reduce CRC incidence and mortality [5]. However, implementation technicalities are often hampering expected screening benefits in many countries [6-8]. Recently, a Canadian expert group investigated the suitability and feasibility of a CRC screening program in Quebec [9]. This group stressed the importance of implementing quality colonoscopy services, which must be on the top of every screening program manager's priority list. Comparable conclusions were achieved by a similar team in New Zealand [10].

Colonoscopy is used for screening and diagnosis of symptomatic or high CRC risk individuals [11-13]. This test allows for direct visualisation of the entire colon, as well as biopsies and excisions of lesions. Colonoscopy requires expertise in order to efficiently identify lesions and perform safe exams [14]. Two requirements are essential to making a test of desired quality. Firstly, the colonoscope must be introduced as far as the proximal end of the colon (the caecum) to enable completeness of the exam. Secondly, the retrieval speed of the colonoscope must be sufficiently slow to allow thorough visual examination of the colon and detection of lesions. A study reported that neoplasia detection rises from 12% to 28% when retrieval time is above six minutes [14]. In addition, a correlation between pace of retrieval and the rate of detection of polyps has also been reported [15,16]. Slowing the pace of retrieval is more beneficial for the detection of small polyps, becoming nonsignificant for polyps of 20 mm and more.

Although considered a standard of reference for adenomas and cancer detection, colonoscopy is not a perfect procedure. Rex *et al*. evaluated the miss rate for adenomas in 183 patients who had two consecutive colonoscopies performed by two different physicians during the same day [17]. The miss rate for adenomas was 24% overall, 27% for adenomas less than 5 mm, 13% for adenomas ranging from 6 to 9 mm, and 6% for adenomas of 1 cm and more. A Manitoba retrospective study of 36,000 individuals without CRC after a colonoscopy showed the limits of this method [18]. After five years, the CRC incidence of this cohort was only 45% lower than that of the general population of the province. Moreover, colonoscopy is less effective for the detection of cancer in the proximal colon than in the distal colon [19-21]. The reasons behind this difference in performance are still ambiguous. Finally, it is worthwhile to mention that a risk of complications does exist, and these complications might be as serious as haemorrhage, perforation, and even death [22-25]. Although relatively rare, these complications can be of great concern in the context of large-scale population screening.

Some interventions have proven effectiveness in reducing the risk of colonoscopy complications. Korman and colleagues investigated the rate of perforations in a very large series (116,000) of colonoscopies performed on outpatients in the United States [26]. All of the 45 clinics in this study where operating under the supervision of a quality assessment committee (QAC) that reviewed all cases with complications and reported them to a Medical Affairs' Officer (MAO). A rate of 0.3 perforations per 1,000 tests was obtained, which is amongst the lowest reported. This study shows that a low rate of perforation is achievable when a large cohort of faecal occult blood test-positive outpatients is being tested in a screening program. A second study conducted in Germany stressed the importance of the level of practice for gastroenterologists [27]. The median number of colonoscopies per physician was 772 (400 to 1200). According to the authors, a high level of practice is associated with a low rate of complications among patients. The preceding two studies demonstrate that CRC screenings of magnitude similar to what is expected in Quebec are feasible and that low rates of complications are also reachable. Although the actual rate of complication in Quebec is unknown, it is unlikely that it is low on account of the lack of QACs reporting to MAOs and the lack of iterative actions that foster practice upgrading of physicians. Identifying and indexing barriers and facilitators to auditing complications and the level of practice of physicians would facilitate implementation of quality colonoscopy services in the healthcare system.

Endoscopist characteristics and the type of setting where the colonoscopy is performed are also related to the quality of this procedure. A population-based study of 110,402 individuals aged 50 to 80 years old from Ontario showed an association between incidence of CRC after a negative colonoscopy and endoscopist speciality [28]. Those who had their colonoscopies performed by a gastroenterologist had a reduced risk of developing subsequent CRC. Bressler and colleagues evaluated the rates of new and missed CRC after colonoscopy and their corresponding risk factors [29]. They found that patients were more likely to have a new or missed CRC when their colonoscopies were performed by family physicians and internists or in office-based practices [29]. In addition, patients who had their colonoscopy done in a private office were more likely to have an incomplete procedure compared with those who had their colonoscopy done in an academic hospital [30]. Finally, endoscopist experience is associated with the risk of complications [31]. Patients who had their colonoscopy performed by a proficient endoscopist had a reduced risk of bleeding and perforation. However, there is no evidence that endoscopist specialty is a risk factor for colonoscopy-related complications [31].

### Challenges to implementing a colorectal screening program in a complex healthcare system

Introduction of CRC screening programs will lead to increased demands for colonoscopy services such that, unless resources are adequately planned, wait times for colonoscopy may be prolonged. For instance, in England [32,33] and Australia [34], where colonoscopy services were used to respond timely to the demands, it was impossible to respond adequately to the surge in demands induced by screening. Several factors hamper accurate estimation of Quebec's healthcare system's ability to meet demands for colonoscopy services [9]. First, available procedures used for monitoring patients and organising appointments are not well established. Second, there are no systems to set priority for demands in colonoscopy. According to the Canadian Association of Gastroenterology, the median time for having a colonoscopy in Quebec is 10 weeks, and 25% of individuals will wait more than 21 weeks [35]. Moreover, 50% of all individuals presenting alarming symptoms will wait more than 6.5 weeks, while another 25% will only have access to a colonoscopy after 16 weeks or more [35]. Therefore, it is certain that Quebec's healthcare system's current ability to meet demands for colonoscopy services is insufficient [9,35]. The appropriateness of colonoscopy is also crucial and must be taken into account when implementing a CRC screening program [9]. Recently, a study evaluated the appropriateness of colonoscopies performed in several European countries and revealed that only 46% are appropriate or necessary [36]. To the best of our knowledge, no similar study has been conducted in Quebec. Consequently, a significant proportion of colonoscopies could be inappropriate, which reduces Quebec's healthcare system's capacity to respond to demands for colonoscopy services and hampers subsequent implementation of a CRC screening program [9]. Nevertheless, it is worthwhile to mention that some interventions, such as education programs, have proven to be effective in reducing the number of inappropriate colonoscopies [37].

It is mandatory to implement an effective and efficient screening program in the healthcare system that is able to reduce CRC incidence and mortality while ensuring a low level of adverse effects [38,39]. In addition, such a screening program must also be reasonable in terms of costs, including medical costs and costs borne by patients, such as time and effort [38]. In the current context of scarce resources, it is a challenge to guarantee the effective use of finite resources allocated to the healthcare system while maintaining quality services [40].

Given the lack of current evidence on effective strategies to implement quality colonoscopy services, there is an urgent need to summarise present knowledge on barriers and facilitators to the implementation of such services.

### Patients and public participation in the implementation of quality colonoscopy services

The public and patients have so far played a marginal role in defining how clinical services could be implemented and used [41]. Nevertheless, they are increasingly calling for greater participation in decisions regarding their health and the future of the healthcare system [42,43]. This desire was recently echoed in a pan-Canadian consultation of healthcare managers, decision makers, researchers, and stakeholders who identified public engagement as one of the key emerging short- and long-term research priorities for the healthcare system [44].

Drolet and colleagues conducted a study to evaluate Quebecers' intention to participate in a CRC screening program [45]. They found that 90% of individuals interviewed will accept a colonoscopy diagnostic test if they have a positive faecal occult blood test, which is a very high proportion [45]. It is essential to highlight that the preceding study evaluated the intention to have a colonoscopy and not the proportion of individuals undergoing a colonoscopy. In fact, studies that evaluated the participation rate in colonoscopy for CRC screening show low levels of participation, even in high-risk individuals [46]. Therefore, taking into account the perspectives of patients and the public in defining quality standards for colonoscopy services appears essential to ensure that the services reflect their values, needs, and preferences. Ultimately, it could increase the participation rate for colonoscopy diagnostic tests and improve health outcomes. Citizens' doubts and concerns should be taken into account in order to achieve the full benefits of healthcare services. In addition, public and patient participation may enlighten us about a number of issues raised by the implementation and use of colonoscopy services, such as informed consent in light of the potential risks and benefits [9].

Patient satisfaction is an important facet to quality colonoscopy services. Considering that the majority of individuals undergoing a screening colonoscopy will not have CRC [47,48], it is important to ensure that the patient's experience of the colonoscopy is acceptable. On the other hand, CRC screening involves surveillance of those who are found to have adenomatous polyps, which represents a large proportion of the eligible population undergoing colonoscopy [49]. In those patients, repeated colonoscopies are required at intervals typically ranging between 3 to 10 years, according to their risk of subsequent advanced neoplasia [49]. The quality of the patient experience has to be acceptable to ensure proper compliance with surveillance guidelines. Aspects of patient-centered colonoscopy care have been developed and disseminated through the National Health Services' Endoscopy Global Rating Scale [50]. This includes timely access to colonoscopy, adequate communication about the procedure, its risks and benefits, the preparation involved for the test, appropriate demeanour of the endoscopist and staff, and ability for patients to provide feedback to the service, as well as the receipt of timely results and well-communicated plan of action [50].

A comprehensive and contextualised literature synthesis is necessary to better understand the needs of the public and patients in pluralist health systems in order to implement quality colonoscopy services.

### Gaps in knowledge addressed by this proposal

Patients' and healthcare professionals' perspectives towards the efficient implementation of quality colonoscopy services have been reported in the literature [41,50-52]. Although a number of countries are now implementing quality colonoscopy services, a knowledge synthesis of barriers and facilitators perceived by healthcare professionals and patients during implementation has not been carried out [45,53]. In a healthcare system that tends towards greater interdisciplinarity [54], it is central to acknowledge the dynamics of each group of users and their interdependence when implementing quality colonoscopy services in a complex healthcare setting.

This knowledge is also central to authorities who wish to level implementation difficulties that could hamper expected benefits from CRC screening. Evidence is urgently needed to prepare for this major shift in our healthcare system and to oversee the factors that could affect its adoption and integration by all potential stakeholders. This project aims at producing knowledge that is relevant, timely, and useful for decision makers who are directly responsible for the optimal implementation of colonoscopy services in all Canadian jurisdictions.

### Goal and objectives

This initiative aims to produce a comprehensive synthesis of actual knowledge and lack thereof on the barriers and facilitators influencing the implementation of quality colonoscopy services. This knowledge will directly inform decision makers on key issues that should be taken into account for the implementation of such services. Our objectives are to (1) conduct a mixed-methods review of the literature on the barriers and facilitators related to the implementation of quality colonoscopy services among different stakeholders (gastroenterologists, surgeons, family physicians, nurses, decision-informants, patients, healthcare services managers, healthcare systems administrators); (2) categorise, synthesise, and compare the perceptions of these different groups; (3) underline the adoption/acceptance factors specific to each group and those specific to collective and interdisciplinary clinical work; and (4) identify strategic issues that need to be addressed in the implementation of quality colonoscopy services in the specific context of the Canadian healthcare system.

## Methods

The guiding principle of this knowledge synthesis is its applicability to answering real challenges faced by decision makers in implementing a CRC screening program. The project is divided into two main phases: reviewing and synthesising relevant literature on barriers and facilitators perceived by all stakeholders to implementation of quality colonoscopy services and validating these findings in the context of the Canadian healthcare system through a Delphi study.

### Phase 1: systematic review of barriers and facilitators to implementation of quality (clinical and patient perspectives) colonoscopy services

In order to achieve objectives 1 and 2, we will conduct a comprehensive review of the scientific literature (qualitative, quantitative, and mixed-methods studies) and other published documentation (technical or *grey *literature) on the various factors that may have an impact on the quality of colonoscopy services. Among these factors, barriers and facilitators to implementation of quality colonoscopy services will be identified. Systematic reviews conducted by the investigators [55,56] and other systematic reviews in the field of healthcare CRC screening will guide the development of search strategies.

### Sources of data

Standardised literature searches will be performed on all relevant databases (MEDLINE, Ovid, Cochrane Central Register for Controlled Trials, Campbell Collaboration Register for Controlled Trials, Current Content, Science Citation Index, Social Sciences Citation Index, LISA, CINAHL, PsycINFO, EMBASE, Electronics and Communications Abstracts, Computer and Information Systems Abstracts, ERIC, ProQuest). The references of the retrieved papers will be reviewed as a potential source of further articles. Other literature will be identified through internet search engines and governments' websites. Publications citing the selected articles as well as other articles from authors of the selected articles will be searched through the ISI Science Citation Index. Finally, specialised email lists will be used to contact experts in the field of CRC screening programs for unpublished studies.

### Inclusion/exclusion criteria

#### Types of studies

Studies must be based on a structured and well-described data collection process; that is, research strategies and measurement tools in relation to the study methodology must be presented. Thus, studies reporting unstructured observations, editorials, comments, or position papers will be excluded. All rigorous quantitative, qualitative, and mixed-methods designs will be considered. Specific scales will be used to assess the quality of each type of design, based on a recent tool that proposes specific criteria for assessing quantitative (experimental and observational), qualitative, and mixed-methods designs [57]. Systematic reviews and meta-analyses will be considered if their main focus is related to barriers and facilitators to implementation of quality colonoscopy services. Studies published in all languages will be included as long as they present an English abstract.

#### Stakeholders in quality colonoscopy services

Stakeholders are gastroenterologists, surgeons, family physicians, nurses, decision-informants, patients, healthcare services managers, and healthcare system administrators, given that they are potentially among the most involved in quality colonoscopy services in healthcare systems [58]. We will consider studies investigating barriers and facilitators to implementation of quality colonoscopy services from the perspectives of patients having already undergone a colonoscopy, as well as the perspectives of precolonoscopy patients. Studies focusing on specific sociocultural groups will be excluded.

#### Intervention

Implementation of quality colonoscopy services will be the targeted intervention. Quality of colonoscopy services is defined as quality and safety (now called Clinical Quality) or consumer care (now called Quality of Patient Experience). We will consider implementation of quality standards related to colonoscopy services.

#### Outcomes

Included studies must clearly mention factors that could be considered to be barriers and/or facilitators to implementation of quality colonoscopy services, and they must include a measurement of quality outcomes.

### Screening and data abstraction

All titles and abstracts will be screened independently by a team consisting of one of the two principal investigators and a research associate to assess fitness of studies with the inclusion criteria. Any discrepancies in study inclusion between the two reviewers will be resolved by discussion with other team members. After retrieval of full text copies of relevant articles, each study will be independently evaluated by two reviewers using specific exclusions and inclusions criteria. For each included study, barriers and facilitators will be categorised and compiled using a validated extraction grid. Stakeholders will be divided into groups of users according to these results. The aforementioned extraction grid has been developed by one of the investigator and combines various factors that are likely to affect healthcare professionals' behaviours identified from existing conceptual frameworks [59,60]. It will be customised to ensure its applicability to studies reporting the perspectives of patients and citizens towards the implementation of quality colonoscopy services.

### Appraisal of study quality and relevance

The quality of all eligible studies will be assessed by two independent reviewers using quality criteria specific to quantitative, qualitative, and mixed-methods designs [61-63]. Studies that fall below a quality threshold on their respective quality scales will be discarded. Any discrepancies in quality ratings will be resolved by discussion and involvement of an arbitrator among other team members if necessary. Studies will be ranked according to their quality. Technical and grey literature will also be appraised for quality, but given that there are no known consensual quality criteria for this type of literature, studies from these sources will be considered to be complementary to the scientific literature.

### Methods for synthesising findings

Findings will be reported using consensual guidelines for narrative syntheses and meta-analytical techniques [60,64-66]. Factors identified will be grouped according to the underlying theoretical concepts. An iterative analytical method will be performed based on transparency and consensus between the reviewers. Thus, other emergent categories of barriers and facilitators might be added to the classification grid during the review process. A narrative synthesis [64,67] will be performed to summarise the evidence from various types of studies according to the quality ranking of studies previously mentioned. A comparison of the barriers and facilitators to implementation of quality colonoscopy services among the various groups represented will be done using meta-analytical techniques. Results will be presented according to each user's group and health consumers for which barriers and facilitators have been studied. In addition, factors that are specific to interdisciplinary clinical work will be clearly identified. This synthesis will provide insight on a wide range of conditions that might influence the acceptance, adoption, utilisation, and integration of quality colonoscopy services in the healthcare system.

### Strategies to ensure methodological rigour

Guidelines from recognised organisations, such as the Cochrane Collaboration, will be followed to ensure the methodological rigour of this systematic review. Given the variability in the nature of the literature that will be assessed through this review, we will make sure that appropriate criteria are used to assess the quality of each type of study (quantitative, qualitative, and mixed-methods).

### Phase 2: pan-Canadian Delphi study

In the second phase of the study (five months), findings from the systematic review of each group of users will be validated for their applicability and their importance to the Canadian context through a Delphi process, involving representatives from each group of users. A Delphi study is a technique that compares the degree of written agreement among participants who are not in contact at anytime [68]. It is considered to be a strong methodology for a rigorous consensus of users on a specific theme. This type of study is highly recommended for obtaining opinions from users who live and work in different geographic areas and settings [69], giving us the opportunity to have a pan-Canadian representation. The anonymity of the Delphi process also encourages open and honest feedback among participants.

### Selection and recruitment of the expert panel

The aim of the Delphi study is to obtain opinions from each group of users representing a variety of expertises and contexts in Canada. As such, 10 to 18 participants [68] for each group of users will be recruited across Canada through professional associations and corporations, regional health authorities, and experts from each province concerned with the implementation of quality colonoscopy services. A list of potential participants will be created through the contacts network method [70], with the help of decision makers of the team and their collaborators. Recruitment of participants will be done through a message sent by email. A postcard will also be sent to allow for contacting participants who do not have email or do not use it, to limit possible selection bias. However, participants have to be able to access the internet to be included in the study. The message will present the study's objectives, the nature of their implications, and will solicit their participation in the Delphi study. The message will also provide a link to the study website (or URL address in the postcard) and give participants a temporary username. Participants will be informed that their participation in the study is entirely voluntary and that they implicitly consent to participate when creating their electronic account.

### The Delphi process

The first step of this Delphi study is to develop a pretest questionnaire from the findings of the systematic review. This questionnaire will focus on the main barriers and facilitators that have been reported in the literature for each group of users. The selection of items will be based on their relative importance in the literature. In general, items mentioned by 15% or more of the studies will be kept, based on content-analysis techniques to identify salient beliefs in the construction of questionnaires [71]. Our advisory committee, representing groups of users, will face validate this questionnaire to ensure the good understanding of the questions and to evaluate the time needed to complete it. After this pretest, a final questionnaire will be prepared and made accessible electronically on the secure website. All potential participants will be sent by mail and email an information sheet about the project, as well as a consent form. After creating their personalized electronic accounts by entering their usernames and choosing their passwords, participants will be guided through the process of the electronic Delphi questionnaire. Participants will be asked to rate the applicability and the importance of each proposed item on a seven-point Likert scale. Results from the first round will be compiled, and a mean score of applicability and importance for each item will be calculated. Then, participants will be invited to partake in a second-round rating process by email and postcard, through the password-protected website [72,73]. Participants will again be asked to rate the degree of applicability and importance of each identified factor. This survey will also show the first-round ratings by providing the mean score for each item. Participants will also be able to add free text comments. Email and postcard reminders will be sent to the participants after each round. A third-round survey, based on the responses of the second round, might be necessary if a consensus is not reached for at least 70% of items [74]. Finally, the consensual rating will be sent a last time to the participants for a final validation.

### Analysis of ratings

Aggregate ratings will be calculated and feedback comments will be content analysed for each round of the survey. In addition, to ensure equal weighting for each user's group in the overall rating, a weighted median will be calculated. A satisfactory degree of consensus will be obtained if less than 30% of the ratings are in the lower range (ratings from 1 to 2) and less than 30% of the ratings are in the upper range (ratings from 6 to 7) [72,73,75].

### Strategies to ensure methodological rigour in the Delphi study

The choice of the items for the Delphi questionnaire will be based on rigorous content-analysis techniques that identify salient beliefs through the number of times mentioned by participants (15% and more will be kept) [71]. In addition, the questionnaire will be face validated by the advisory committee that represents groups of users. Finally, to ensure that the panel of participants represents specialists recognised by their peers, the selection of experts will be done with the help of members of the advisory committee who are in strategic positions to give this information (healthcare professional associations and corporations). The invitation to participate in the Delphi study will be sent to 30 participants of each group to ensure that at least 10 of them will answer all the rounds of the study. These invitations will be sent by mail and email to limit possible selection bias by allowing for contacting participants who do not have email or do not use it.

### Ethical considerations

Exemption from ethics approval has been received from the Research Ethics Board of the Centre Hospitalier Universitaire de Québec Research Centre (11 August 2010; ethics number S10-09-067-AQEM). Participants to the Delphi study will be sent emails presenting the study's objectives and information about research implications. They will be informed that their participation in the study is entirely voluntary and that they implicitly consent to participate when creating their electronic account.

## Discussion and implications

This study will produce a comprehensive synthesis of actual knowledge on the barriers and facilitators perceived by stakeholders to implementation of quality colonoscopy services. Moreover, the Delphi study will validate findings from the literature review, which constitutes a novel approach that produces results that are more attractive for decision makers and more directly applicable to the elaboration of policies and strategies in the Canadian context. The results of this study will be particularly relevant, timely, and useful for decision makers who currently face the challenge of implementing quality colonoscopy services and CRC screening programs in the healthcare system.

In addition, this project will favour partnerships between researchers and direct stakeholders of research results (strategic collaborators and decision makers) by integrating them in all steps of the project and integrating their specific needs in the way that the knowledge is synthesised, transferred, and applied. The participation of strategic decision makers in the research team will encourage knowledge sharing through their networks. These sustained relationships between all members of the team and collaborators will greatly contribute to the relevance, uptake, and utilisation of the results to support an optimal implementation of quality colonoscopy services in the Canadian healthcare system.

In conclusion, this study will be the first to systematically summarise the barriers and facilitators (clinical and patients' perspectives) to implementing quality colonoscopy services perceived by different groups and to consider the local contexts in order to ensure the applicability of this knowledge to the particular realities of the various Canadian jurisdictions. Linkages with strategic partners and decision makers in the realisation of this project will favour the utilisation of its results to support strategies for implementing quality colonoscopy services in the Canadian health system.

## Competing interests

The authors declare that they have no competing interests.

## Authors' contributions

All authors collectively drafted the research protocol and approved the final manuscript.
